# Sex-specific NLRP3 activation in neutrophils promotes neutrophil recruitment and NETosis in the murine model of diffuse alveolar hemorrhage

**DOI:** 10.3389/fimmu.2024.1466234

**Published:** 2024-11-25

**Authors:** Pierre-André Jarrot, Jiyoun Kim, William Chan, Lukas Heger, Nicolas Schommer, Pierre Cunin, Camila M. S. Silva, Stéphane Robert, Peter A. Nigrovic, Bruce Ewenstein, Denisa D. Wagner

**Affiliations:** ^1^ Program in Cellular and Molecular Medicine, Boston Children’s Hospital, Boston, MA, United States; ^2^ Department of Pediatrics, Harvard Medical School, Boston, MA, United States; ^3^ Department of Cardiology and Angiology, University Hospital of Freiburg Bad Krozingen, Freiburg, Germany; ^4^ Department of Cardiology, Angiology, Haemostaseology and Medical Intensive Care, University Medical Center Mannheim Medical Faculty Mannheim, Heidelberg University, Mannheim, Germany; ^5^ Division of Immunology, Department of Pediatrics, Boston Children’s Hospital, Harvard Medical School, Boston, MA, United States; ^6^ Aix-Marseille Univ, INSERM (National Institute of Health and Medical Research), INRAE (France’s National Research Institute for Agriculture, Food and Environment), C2VN (Center of Cardiovascular Research and Nutrition), AMUTICYT, Marseille, France

**Keywords:** NLRP3 inflammasome, neutrophil extracellular traps, diffuse alveolar hemorrhage, sexspecific, murine model

## Abstract

**Objectives:**

Diffuse alveolar hemorrhage (DAH) is a life-threatening complication of systemic lupus erythematosus and small vessel vasculitis. We previously showed that neutrophil extracellular traps (NETs) were associated with the pathogenesis of pristane-induced DAH and demonstrated that neutrophil NOD-like receptor family pyrin domain containing 3 (NLRP3) inflammasome assembly participated in NET generation under sterile stimulation. We investigated whether NLRP3 inflammasome assembly in neutrophils may drive pulmonary NETosis in a mouse model of pristane-induced DAH.

**Methods:**

C57BL/6J mice received a single intraperitoneal injection of 0.5mL of pristane. Neutrophil NLRP3 inflammasome assembly and NETs were characterized by immunofluorescence staining of apoptosis-associated speck-like protein a CARD (ASC), co-staining of DNA, and citrullinated histones, respectively. Clinical status of mice was assessed 11 days after pristane injection by measurement of arterial oxygen saturation and of weight loss; severity of lung injury was determined using a quantification score from hematoxylin-eosin-stained slides.

**Results:**

Pristane induced ASC speck formation in neutrophils and we confirmed that NLRP3 inflammasome was involved in NET generation after pristane stimulation *in vitro*. NLRP3 deficiency reduced the severity of pristane-induced DAH in female, but not male mice. Interestingly, NLRP3 deficiency reduced the number of neutrophils and NETs in the lungs of females compared to males.

**Conclusions:**

Our results suggest a link between female sex-specific NLRP3 inflammasome activation and subsequent pulmonary NETosis in the development of pristane-induced DAH. Therefore, we identified NLRP3 inflammasome as a potential new therapeutic target in this severe complication of pro-female autoimmune disease for which specific inhibitors of NLRP3 are currently developed.

## 1 Introduction

Diffuse alveolar hemorrhage (DAH) is characterized by a leakage of red blood cells from lung capillaries in the alveoli. The prognosis is severe, with a 20% mortality rate due to limited therapeutic options. Autoimmune diseases represent 35% of cases of DAH, in which systemic lupus erythematosus (SLE) and small vessel vasculitis (SVV) are the main etiologies (1). Pathological examination of lungs reveals hemosiderin-laden macrophages, bland hemorrhage, and neutrophils interstitial infiltrates, which constitute pulmonary capillaritis, the hallmark of small vessels vasculitis (2). However, the molecular mechanisms responsible for the development of these conditions remain elusive, and there are no Food and Drug Administration-approved drug therapies so far.

NLR family pyrin domain containing 3 (NLRP3) is the most characterized intracellular sensor that detects pathogen-associated molecular patterns (PAMPs) and damage-associated molecular patterns (DAMPs). Upon its activation, apoptosis-associated speck-like protein containing a CARD (ASC) self-associate into a helical fibrillary polymer, resulting in the formation of the ASC speck, acting as a molecular platform for pro-caspase-1 via proximity-induced auto-catalytic activation (3). Caspase-1 is critical to the inflammatory response by enzymatically cleaving pro-interleukine-1 (pro-IL-1β) and pro-IL-18 into their bioactive forms (IL-1β and IL-18) and gasdermin D to promote lytic cell death called pyroptosis. Besides macrophages, neutrophils have also been described as a source of NLRP3/ASC-dependent IL-1β production after *Staphylococcus aureus* infection or sterile thioglycolate-induced peritonitis (4, 5). Our group previously demonstrated that NLRP3 deficiency reduced neutrophil recruitment in the inflamed peritoneal cavity, suggesting that NLRP3 participated in neutrophil recruitment (5). Interestingly, we showed that peptidylarginine deiminase 4 (PAD4), critical for NETosis, supported NLRP3 inflammasome assembly and promoted NETosis under sterile conditions *in vitro* and *in vivo* in a deep vein thrombosis murine model (6).

Pristane is also known as hydrocarbon oil (2, 6, 10, 14-tetramethylpentadecane), found in small quantities in many plants, the liver of sharks, and as a byproduct of petroleum distillation (7). Intraperitoneal (IP) injection of pristane has a pro-inflammatory effect, inducing recruitment of neutrophils and monocytes to the peritoneal cavity leading to a sterile peritonitis (8). Another study suggested procoagulant and prothrombotic properties since inadvertent cutaneous injection of pristane in humans working in shipyards induced skin necrosis (9). Pristane-induced DAH is a well-known model considered to be the first step of pristane-induced lupus (10). In the first 15 days after IP injection of pristane, C57BL/6 mice develop severe DAH with similar pathological lesions to DAH-related autoimmune diseases. A pathogenic role of neutrophils was suspected since their recruitment in lungs preceded hemorrhage, starting 3 days after pristane injection, and peaking at 2 weeks. In addition, the combination of the detection of NETs in the lungs of pristane-induced DAH mice and the improvement of their clinical status with subsequent reduced lung injury after deoxyribonuclease-1 aerosol therapy, which is known to disrupt NETs’ scaffold, suggested a role of neutrophils and NETs in the development of the lung injury (11).

Here, we tested the hypothesis that neutrophil NLRP3 inflammasome activation plays a role in the pathogenesis of pristane-induced DAH by promoting pulmonary NETosis. We showed that pristane-induced neutrophil NLRP3 activation participated in NET formation *in vitro*. Genetic deletion of NLRP3 conferred less severe DAH in females, with lower recruitment of neutrophils and NET generation in lungs, compared to males.

## 2 Methods

### 2.1 Animals

Both male and female C57BL/6J NLRP3-/- (stock no. #021,310) and age- and sex-matched wild type (WT) C57BL/6J (NLRP3 +/+) (stock no. #000,652) mice were obtained from Jackson Laboratory (Bar Harbor, ME, USA). All mouse lines were housed in the animal facility of Boston Children’s Hospital (Boston, USA). All experimental animal procedures were approved by the Institutional Animal Care and Use Committee of Boston Children’s Hospital under the protocol number 20-2-4097R. Each experiment had n=3-6 mice/control group and n=7-10 mice/treated group.

### 2.2 Murine neutrophil isolation

Peripheral blood from males and females was collected from the retro-orbital vein using the heparinized capillary tube from NLRP3-/- or NLRP3+/+ mice. One mL of blood was obtained from isoflurane (2% inhaled) anesthetized mice through the retroorbital plexus into 2 mL of preheated (37°C) anticoagulant buffer (15 mM EDTA and 1% endotoxin free bovine serum albumin in sterile PBS). Mice were then submitted to deep inhalation with isoflurane (4%) before cervical dislocation to ensure euthanasia. The blood was centrifuged at 500 g for 12 min at room temperature, after which the supernatant was removed, and cells were resuspended in anticoagulant buffer before being loaded on top of a 3-layer Percoll gradient column of 78%/69%/52% in a 15-mL centrifuge tube. After loading the blood cells on top of the 52% layer, the column was centrifuged in a swinging bucket centrifuge for 32 min at 1,500 g at room temperature, with acceleration set at 3, and brake at 0. Cells at the 69%/78% interface were collected, washed, and pelleted by adding sterile PBS and centrifuged for 12 min at 500 g. After lysis of red blood cells with ammonium-chloride- potassium lysis buffer, cells were resuspended in imaging media (phenol red-free RPMI 1640 supplemented with 10 mM N-2- hydroxyethylpiperazine- N′- 2- ethanesulfonic acid) and further used for experimental procedures.

### 2.3 *In vitro* ASC speck and NET visualization

Freshly isolated neutrophils were either used directly or pretreated with MCC950 (Invivogen, inh-mcc), a specific inhibitor of NLRP3 inflammasome. Neutrophils were incubated with 1μM of MCC950 or vehicle for a period of 30 min at 37°C and 5% CO^2^. After treatment, neutrophils were washed, pelleted, and resuspended before stimulation. The isolated neutrophils were allowed to adhere to a sterile coverslip in a 24-well plate for 30 min at 37°C and 5% CO_2_ in a concentration of 6 x 10^5^/mL. The attached neutrophils were either incubated with ionomycine (4µM), used as a positive control for NLRP3 activation and for NETosis, or incubated with pristane complex with β-cyclodextrin (BCD (26.6µM) Pristane (13.3µM)) (Sigma-Aldrich, P2870). Generation of BCD pristane complex resulted from a 4mM solution of BCD mixed with pristane (2mM final concentration) and stirred for 4 days at room temperature, as described previously (12). All complexes were prediluted at 1/150 in PBS before making experiments.

Neutrophils were stimulated for one hour at 37°C, 5% CO_2_ to assess ASC speck formation and NET generation, respectively. After stimulation, neutrophils were fixed by adding 2% paraformaldehyde (PFA) for 15 min at room temperature, permeabilized using 0.1% of triton X-100 in PBS-/- for 10 min at 4°C, and blocked with blocking buffer (2,5% BSA, 0,5% Tween-20 in PBS) during one hour at 37°C.

Cells were incubated overnight at 4°C with primary rabbit polyclonal antibody (Ab) directed against ASC (1:500, Cell Signaling, D2W8U) or H3 citrullinated histones (H3-Cit) (1:500, Abcam, ab5103) and subsequently incubated with rabbit-specific secondary 488-AlexaFluor dye Ab (1:1500, ThermoFischer) for 2h at room temperature. Cells were counterstained using Hoeschst 33342 (1:10,000, Invitrogen) for 10 min at room temperature and the coverslips were mounted and visualized for ASC speck formation and NET formation. Images were acquired using a Keyence BZ-X810 microscope equipped with a 60x oil-emersion lens and processed with FIJI/Image J software (NIH, USA).

ASC speck formation and NET quantification were performed in a blinded manner. Cells were washed 3 times with PBS-/- at each step of staining until the mounting final step. ASC speck frequency was determined by capturing 10 random microscopic fields per condition. NET formation was quantified using a modified method previously reported (13). Briefly, using the 20x objective, the percentage of neutrophils releasing NETs was quantified in sample by assessing the fraction of neutrophils releasing DNA fibers with H3-Cit co-staining. NET percentage was calculated as follows: NET rate (%) = 100 x number of neutrophils releasing DNA fibers with H3Cit co-staining/total number of neutrophils. Quantification was performed using 2 delimited standardized plots evaluating 10 random microscopic fields per condition. An average number of 25 neutrophils were analyzed in each field of view.

### 2.4 Pristane-induced DAH model

Mice received a single IP injection of 0.5mL synthetic sterile-filtered liquid pristane (Sigma-Aldrich, P2870). Control mice received a 0.5mL PBS IP injection. The final stage was on day 11 after pristane injection where real-time oxygen-saturated hemoglobin (percentage of functional arterial hemoglobin) was measured before euthanasia using a MouseOx pulse-oximeter (Starr Life Sciences). Hairs from the collar region (back of the neck) were removed using a depilatory agent 5 days before actual measurement. A disposable sensory collar clip attached to the pulse-oximeter was placed on the hairless area, and measurements were initiated through MouseOx software (version 6.3; provided by the manufacturer). Recorded values during a 10-minute consecutive interval were pooled for each mouse, and median values were then used for analysis.

### 2.5 Bronchoalveolar lavage

BAL was carried out as described earlier at day 11 (14). Briefly, mice were euthanized by IP injection of sodium pentobarbital (100mg/kg) solution in PBS and the trachea was cannulated. BAL fluids were collected with 2 separate 1mL cold PBS/100μM EDTA washes of lung via the trachea of each mouse. Fluids were washed for 7 min at 400 x g, 4°C. Cell pellet was resuspended in 200μL of ACK lysing buffer for 2 min at room temperature. Cells were centrifuged for 7 min at 400 x g, 4°C, and resuspended in 200μL, BSA 0.5%.

### 2.6 Fluorescence cytometry

Fc receptors were blocked with TruStain™ (anti-mouse CD16/32, Biolegend)) for 10 min at room temperature and washed in PBS/BSA 0.5%. Cells were immunostained with anti-mouse CD45 APC CY7 (1/100, Biolegend, 157204), anti-mouse Ly6C APC (1/100, Biolegend, 128016), anti-mouse Ly6G FITC (1/200, Biolegend, 127605), and F4/80 PE (1/100, Biolegend, 123110) Ab for 10 min at room temperature, dark. Cells were washed and resuspended in 200μL of PBS/BSA 0.5% to get the concentration of neutrophils. We added to the immunostained sample an equivalent volume of counting fluorescent beads (Biolegend, 424902) whose concentration is provided by the manufacturer and stable over the time. Beads were detected by the instrument (LSR Fortessa X-20, Becton Dickinson) and gated based on their scatter and fluorescence properties. To calculate neutrophil concentration, we applied the following formula:

[Neutrophil concentration = (events neutrophils x initial concentration of beads)/events counting beads].

### 2.7 Pathological investigations

Mice were submitted to deep inhalation with isoflurane (4%) before cervical dislocation to ensure euthanasia. Lung tissue was harvested immediately after euthanasia. The left lobes were fixed in 10% formalin (Sigma-Aldrich) and embedded in paraffin. The right lobes were frozen with optimal cutting temperature (OCT) compound (Sigma-Aldrich). Paraffin-embedded tissue was cut into 5 μm sections and stained with hematoxylin-eosin (H&E) (Sigma-Aldrich). DAH was classified into 4 degrees of severity according to the percentage of hemorrhage on H&E-stained sections as assessed by two blinded independent investigators to determine the DAH score as follows: (0) No hemorrhage, (1) 0 – 25%, (2) 25 – 50%, (3) 50 – 75% and (4) 75 – 100% of hemorrhage.

OCT-embedded frozen lung lobes were cut into 5 μm sections, fixed in 4% PAF for 30 min at room temperature, permeabilized with 0.1% of triton X-100 in PBS-/- for 10 min at 4°C, and blocked with blocking buffer (2,5% BSA, 0,5% Tween-20 in PBS) during one hour at 37°C. Sectioned slides were washed three times with PBS -/- at each step of the experiment. Sections were then incubated with FITC rat anti-Ly6G Ab (1:250, 127605, Biolegend) and rabbit polyclonal anti-H3Cit (1:200, Abcam, ab5103) Ab overnight at 37°C. Sections were then treated with rabbit-specific secondary 649-AlexaFluor dye Ab (1:1500, ThermoFischer) for 2h at room temperature. Cells were counterstained using Hoechst 33342 (1:10,000, Invitrogen), 10 min, at room temperature. Neutrophil count was performed at ×20 magnification in 3 random microscopic fields of each lobe per mouse, quantification of H3-Cit was assessed in the same microscopic fields, followed by binary analysis using Image J 1.49v software (NIH).

### 2.8 Lung intravital microscopy

Images were obtained with a two-photon microscope Thorlabs Bergamo II equipped with a Chameleon Discovery NX laser. Images were obtained with the laser tunable line set at 850 nm (for the lung). Mice were anesthetized with ketamine (100mg/kg) and xylazine (10mg/kg), secured with tape to a custom heated microscope stage and a tracheostomy was performed. The tubing was adapted to a rodent ventilator to facilitate mechanical ventilation (model 845 - Harvard Apparatus). Mice were ventilated with pressure control ventilation (12–15 cmH2O), a respiratory rate of 115 breaths per minute, FiO2 of 0.5–1.0, and PEEP of 3 cmH2O. Isoflurane was continuously delivered at 2% to maintain anesthesia. The mice were then placed in the right lateral decubitus position and the thorax was exposed by removing the skin and fatty tissue above the thorax using sterile forceps and scissors. An incision between two left anterior ribs was performed and the left lung was carefully exposed. An imaging apparatus (which consists in a thoracic suction window (obtained from Mark Looney at UCSF (15)) was attached to a micromanipulator on the microscope stage, placed into position, and 25–35 mmHg of suction was applied (Amvex Corporation) to gently immobilize the lung. The two-photon microscope objective Olympus XLUMPLFLN 20X water dipping objective was then lowered into place over the 12 mm coverslip on the thoracic suction window. Anesthetized mice were injected with a rat anti-Ly6G BV421 (Biolegend, 127627, 20μg) and Dextran Rhodamine B (D1824, 10μL) and fields of views were imaged for 7min, 1 image/second at a resolution of 512x512. Neutrophil count was performed in 3 random microscopic fields per mouse. Three mice were evaluated in the control and the treated group.

### 2.9 Statistics

All experiments were performed in triplicate. All data, except prevalence of DAH, are represented as median and interquartile range (IQR). Statistical analysis was performed using GraphPad Prism (9.3.1). Significance was tested through Mann-Whitney *U* test when comparing two groups, and through ANOVA multiple comparison test for the comparison of more than two groups. P<0.05 was considered statistically significant.

## 3 Results

### 3.1 Pristane-induced NLRP3 inflammasome activation participates in NET formation *in vitro*

**Figure 1:**
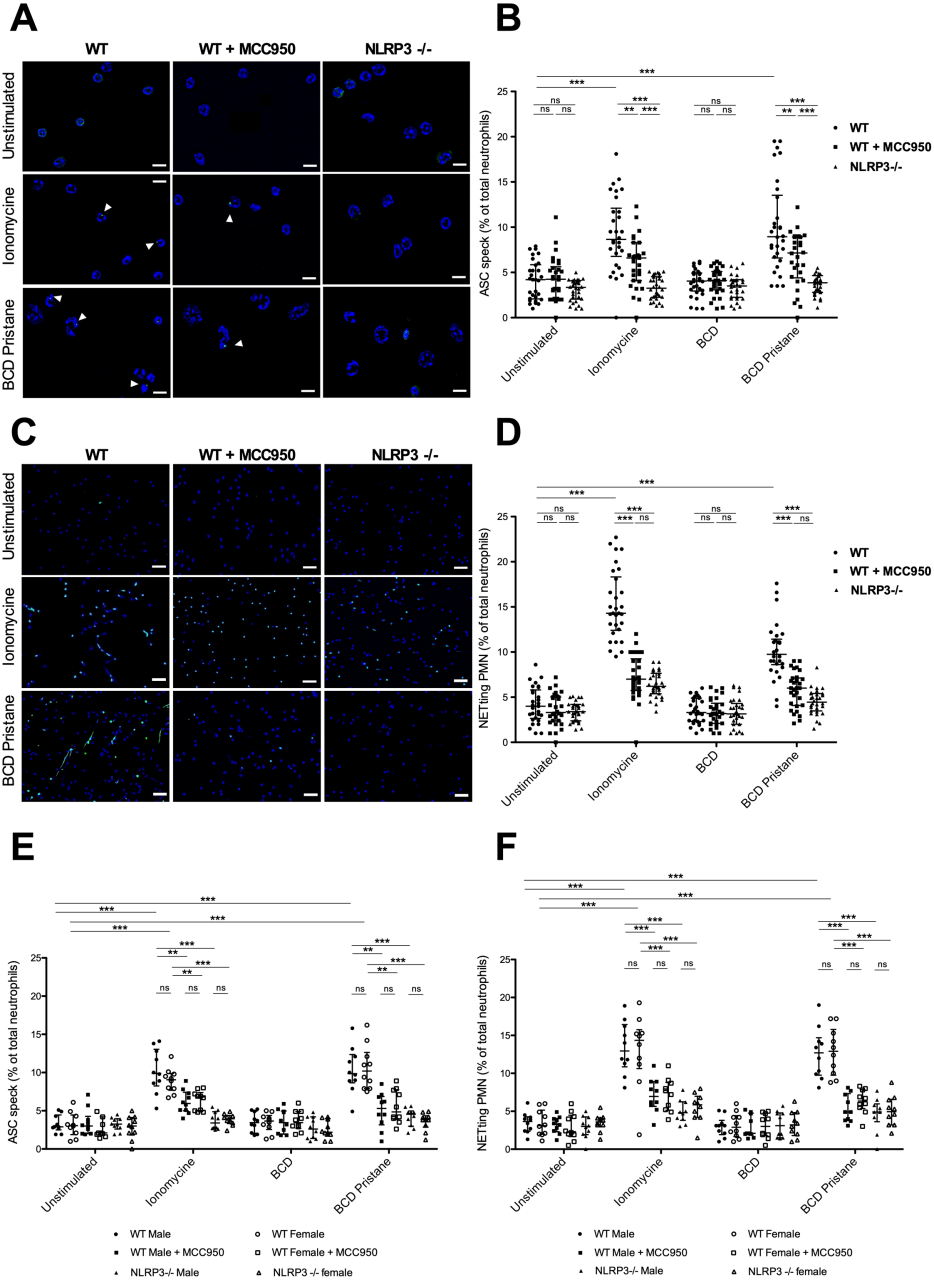
Pristane-induced NLRP3 inflammasome activation promotes NET formation *in vitro* **(A)** Representative IF images of ASC speck formation in isolated murine WT neutrophils, WT neutrophils + MCC950 and NLRP3-/- neutrophils either unstimulated or stimulated with 4µM of ionomycine or with β-cyclodextrin-pristane (BCD pristane): staining of DNA (blue) and ASC (green). White arrows show cells with ASC speck. Stimulation of neutrophils with ionomycine was a positive control for inflammasome formation. Bar 20µm. **(B)** Quantification of ASC speck formation in WT neutrophils, WT neutrophils + MCC950 and NLRP3-/- neutrophils either unstimulated or stimulated with 4µM of ionomycine, with BCD or with BCD pristane. Results represent median ± interquartile range, ANOVA multiple comparison test was used for statistical analysis between groups (ns: non-significant*, p* < 0.01^**^, *p* < 0.001^***^). **(C)** Representative IF images of NETs (colocalization of DNA and extracellular citrullinated histones (H3-Cit) in WT neutrophils, WT neutrophils + MCC950 and NLRP3-/- neutrophils either unstimulated or stimulated with 4µM of ionomycine or BCD pristane: staining of DNA (blue) and H3-Cit (green). Bar 75µm. **(D)** Quantification of NETs released in WT neutrophils, WT neutrophils + MCC950 and NLRP3-/- neutrophils either unstimulated or stimulated with 4µM of ionomycine, with BCD or with BCD pristane. NETs were identified as neutrophils releasing DNA fibers with H3-Cit co-staining. Results represent median ± interquartile range, ANOVA multiple comparison test was used for statistical analysis between groups (ns: non-significant*, p* < 0.001^***^) **(E)** Quantification of ASC speck formation in neutrophils obtained from male or female mice as indicated in the figure: WT neutrophils, WT neutrophils + MCC950 and NLRP3-/- neutrophils either unstimulated or stimulated with 4µM of ionomycine, with BCD or with BCD pristane. Results represent median ± interquartile range, ANOVA multiple comparison test was used for statistical analysis between groups (ns: non-significant*, p* < 0.01^**^ *p* < 0.001^***^). **(F)** Quantification of NETs released in neutrophils obtained from male or female mice as indicated in the figure: WT neutrophils, WT neutrophils + MCC950 or NLRP3-/- neutrophils either unstimulated or stimulated with 4µM of ionomycine, with BCD or with BCD pristane. NETs were identified as neutrophils releasing DNA fibers with H3-Cit co-staining. Results represent median ± interquartile range, ANOVA multiple comparison test was used for statistical analysis between groups (ns: non-significant*, p* < 0.001^***^).

We tested whether NLRP3 inflammasome activation is involved in NET formation after pristane stimulation. We first sought to determine whether *in vitro* stimulation of neutrophils with pristane resulted in speck formation in WT, WT pretreated with MCC950, and NLRP3 -/- cells (**Figures 1A, B**).

MCC950 is a specific inhibitor of NLRP3 inflammasome that directly targets the NLRP3 NATCH domain which is crucial for adenosine triphosphate (ATP) binding, a requirement for NLRP3 oligomerization (16).

Neutrophils were fixed and immunostained for ASC after stimulation with either ionomycine or BCD-pristane. Because of its extreme hydrophobicity, pristane was complexed with BCD. BCD is an oligomer of D-glucose with a hydrophobic cavity and a hydrophilic surface facilitating an effective delivery of pristane. The dose of pristane chosen in our experiment was adjusted in accordance with a previous study on DAH model (11). Levels of ASC-speck formation were increased in WT neutrophils when stimulated with either ionomycine or BCD-pristane compared to WT pretreated with MCC950 and NLRP3-/- neutrophils exposed to similar conditions. Moreover, levels of ASC-speck formation were less important in NLRP3-/- neutrophils compared to WT pretreated with MCC950 neutrophils. We then assessed whether NLRP3 inflammasome formation was required to promote NETosis from pristane-stimulated neutrophils (**Figures 1C, D**). Using fluorescence microscopy, NET formation was determined by the colocalization of DNA fibers and extracellular H3-Cit proteins. WT neutrophils pretreated with MCC950 and NLRP3 -/- neutrophils showed significantly decreased NETosis after ionomycine or BCD-pristane stimulation compared with WT neutrophils. In addition, levels of ASC speck formation and NETosis were similar between male and female neutrophils exposed to similar conditions (**Figures 1E, F**). Taken together, these results showed that NLRP3 was needed for speck formation and NLRP3 inflammasome activation participated in pristane-induced NETosis *in vitro* independently of the sex of mice from which they were isolated.

### 3.2 NLRP3 deficiency reduces severity of pristane-induced DAH in female, but not male mice

**Figure 2:**
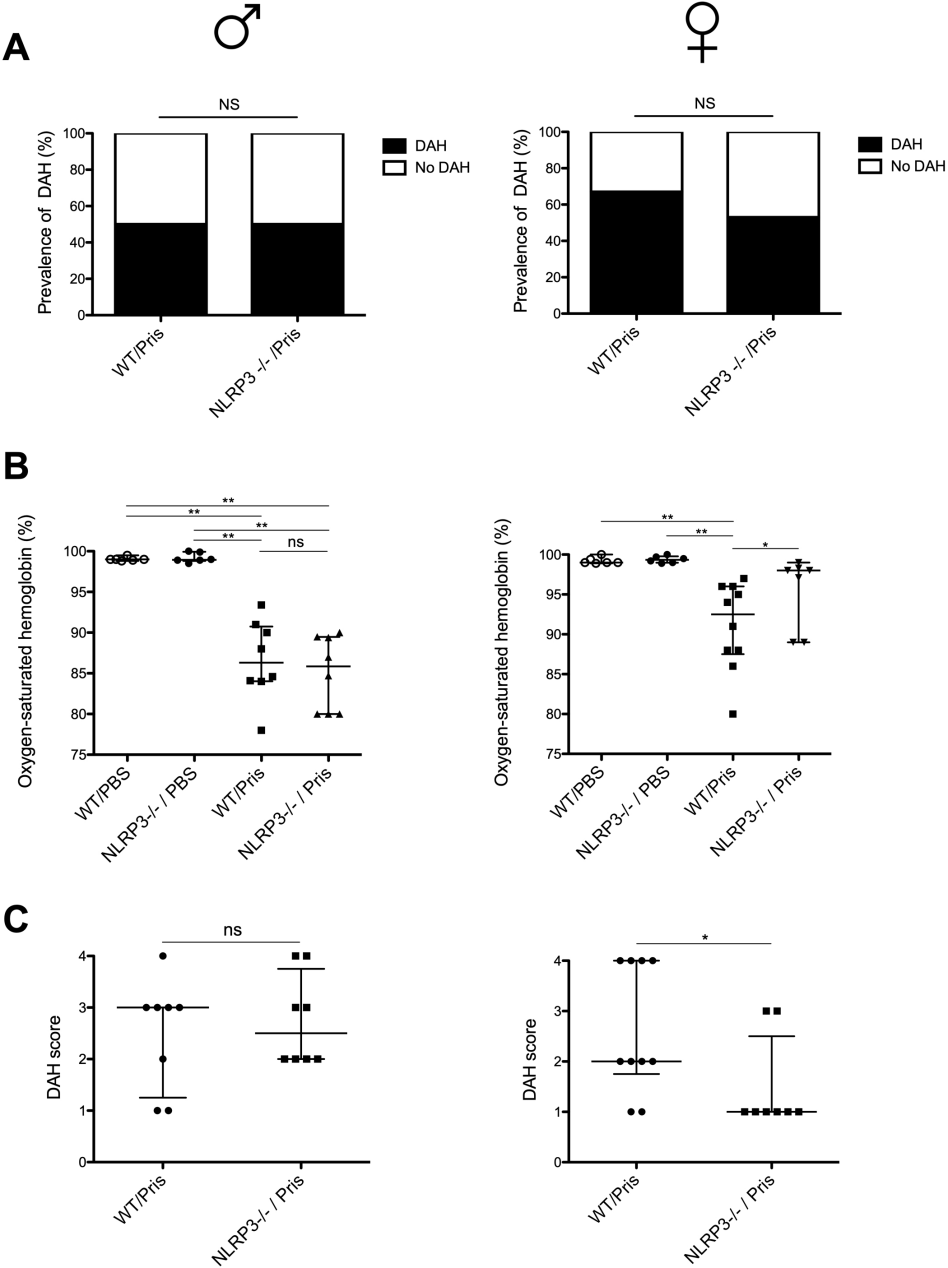
NLRP3 deficiency reduces severity of pristane-induced DAH in female, but not in male mice **(A–C)** Left column shows results with male mice (♂), right with female mice (♀). **(A)** Prevalence of pristane-induced DAH in WT and NLRP3 -/- mice, Mann Whitney test was used for statistical analysis to compare the two groups (ns: non-significant) **(B)** Oxygen-saturated hemoglobin level (%) in mice with pristane-induced DAH WT and NLRP3 -/- mice. WT and NLRP3-/- mice challenged with PBS IP injection were used as negative control. Results represent median ± interquartile range, ANOVA multiple comparison test was used for statistical analysis between groups (ns: non-significant, *p* < 0.05^*^, < 0.01^**^) **(C)** DAH score from pristane-induced DAH WT and NLRP3 -/- mice. Results represent median ± interquartile range, Mann Whitney test was used for statistical analysis to compare the two groups (ns: non-significant, *p* < 0.05^*^). Each experiment had n=3-6 mice/PBS group and n=7-10 mice/pristane group.

**Figure 3:**
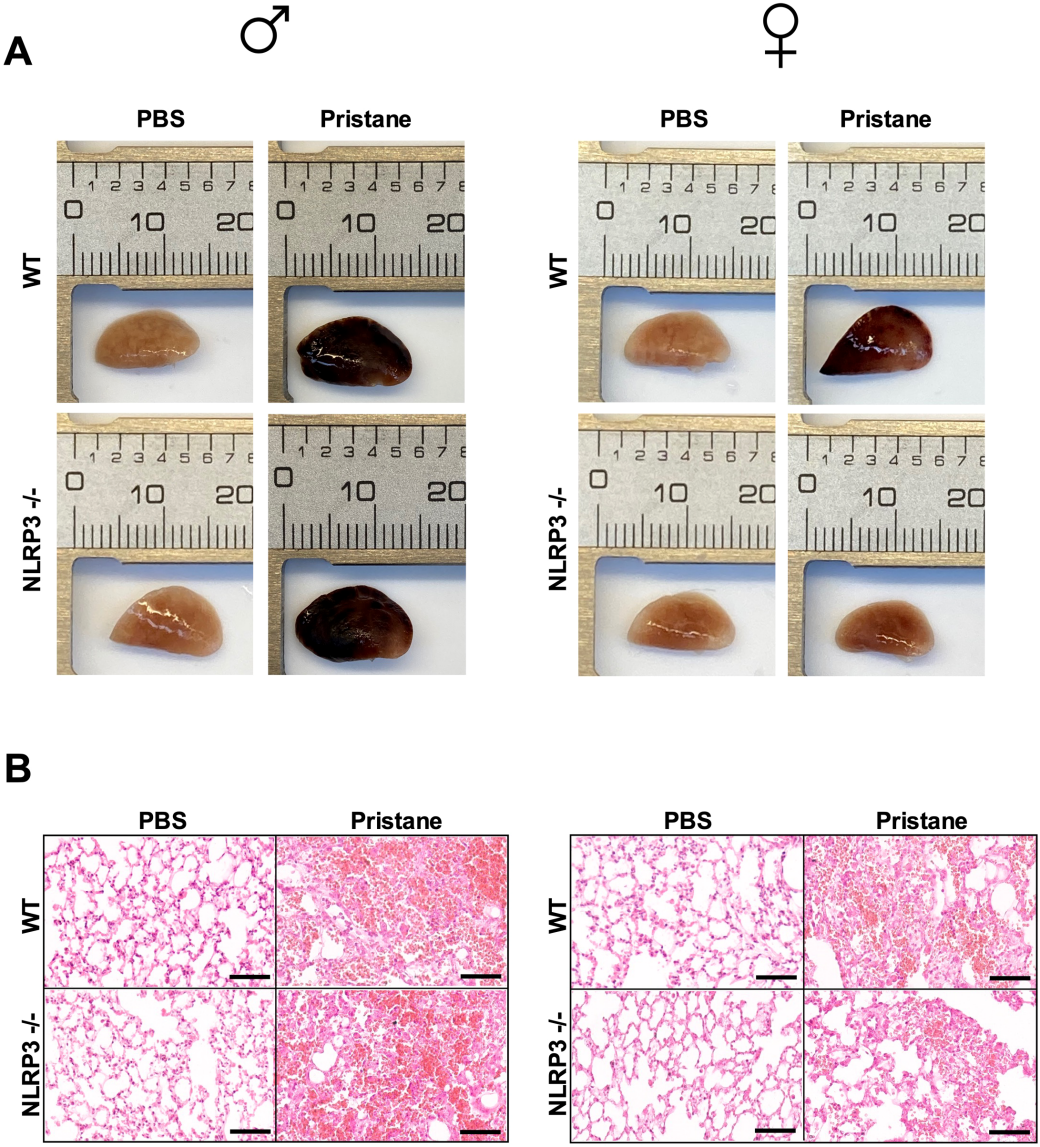
NLRP3 deficiency reduces severity of pristane-induced DAH in female, but not in male mice, representative macroscopic and microscopic views of lungs. **(A, B)** Left column shows results with male mice (♂), right with female mice (♀). **(A)** Macroscopic view of the left lung lobe from PBS group and pristane-induced DAH group. **(B)** Representative H&E-stained section of left lung lobe from pristane-induced DAH WT and NLRP3 -/- mice. Bar 100µm. WT and NLRP3-/- mice challenged with PBS IP injection served as negative control.

We investigated whether NRLP3 deficiency could have an impact on the prevalence and the severity of DAH in both sexes. Severity was determined by measuring lung respiratory function and DAH pathological score (**Figures 2A–C**). Severity of DAH was similar between WT males and WT females (**Supplementary Figure 1**). Prevalence of DAH was similar between WT and NLRP3-/- mice as well as between male and female mice (**Figure 2A**) However, in pristane-injected female NLRP3 -/- mice, arterial oxygen-saturated hemoglobin was higher (**Figure 2B**) and DAH pathological score was reduced when compared to injected WT female mice (**Figure 2C**). In contrast, in pristane-injected male NLRP3-/- mice, no significant differences were seen compared to injected WT male mice. Lung macroscopic and H&E-stained microscopic views are presented in **Figures 3A, B**. In addition, no significant difference was detected on the maximum weight loss and the wet/dry ratio between NLRP3-/- and WT animals in both sexes (**Supplementary Figure 1**). These data suggested that NLRP3 deficiency reduced the severity of pristane-induced DAH in female, but not male mice.

### 3.3 NLRP3 deficiency reduces number of neutrophils and citrullinated histones in lungs of pristane-induced DAH females, but not males

**Figure 4:**
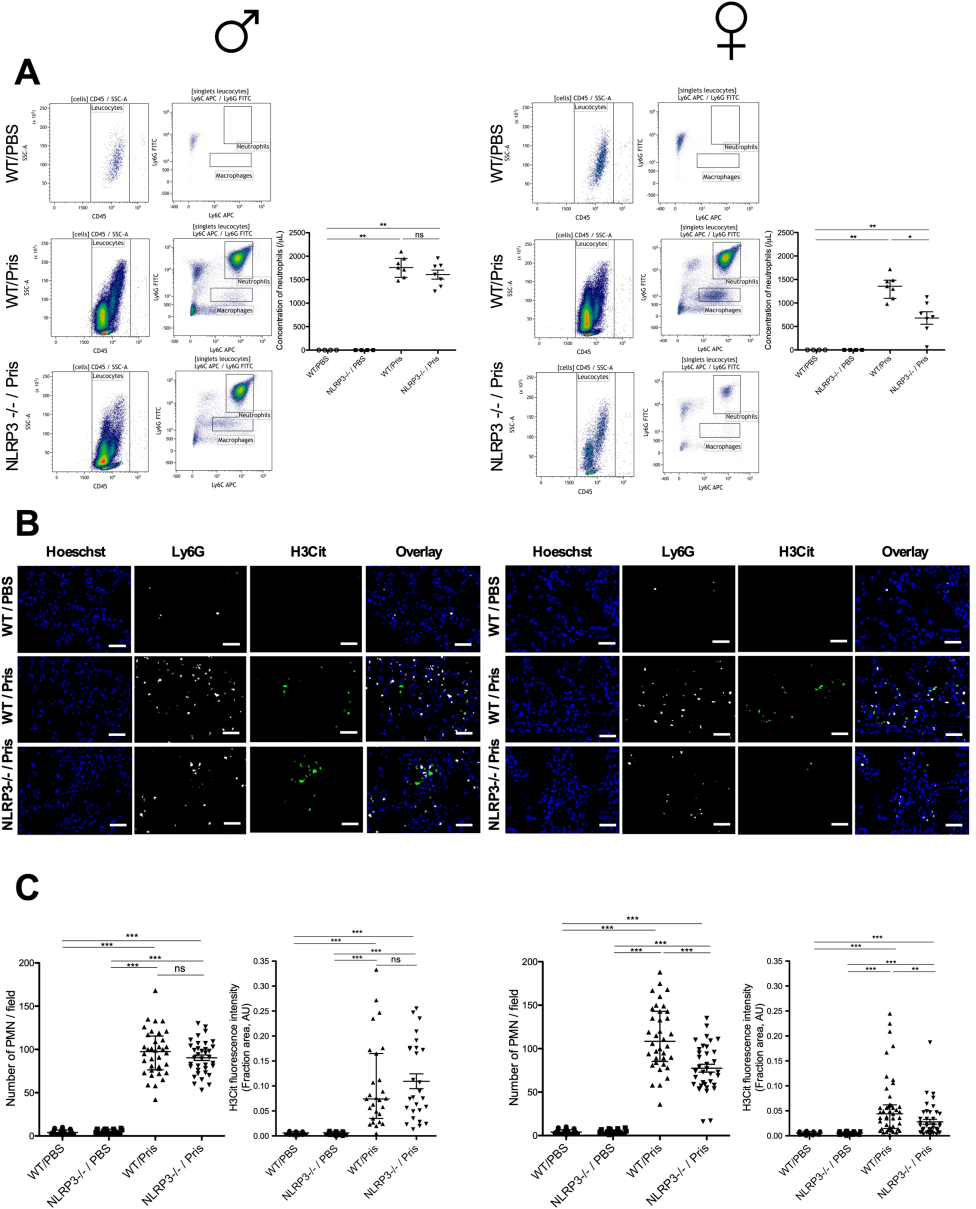
NLRP3 deficiency reduces number of neutrophils and amount of NETs in lungs of pristane-induced DAH female, but not in male mice **(A–C)** Left column shows results with male mice (♂), right with female mice (♀). **(A)** Flow-cytometry analysis of BAL from pristane-induced DAH WT and NLRP3 -/- male and female mice. WT and NLRP3 -/- males and females challenged with PBS IP injection were used as negative control. All cells were CD45+, neutrophils were identified as Ly6C+/Ly6G+, macrophages were identified as Ly6C+/Ly6G-. Quantification of neutrophils in BAL from pristane-induced DAH WT and NLRP3 -/- male. WT and NLRP3 -/- male challenged with PBS IP injection were used as negative control. Results represent median +/- interquartile range, ANOVA multiple comparison test was used for statistical analysis between groups (ns: non-significant, *p* < 0.05^*^, *p* < 0.01^**^). **(B)** Lung tissue cryosections were obtained from pristane-induced DAH WT and NLRP3 -/- mice. WT and NLRP3 -/- mice challenged with PBS IP injection were a negative control: cryosections were immunostained for DNA (blue), Ly6G (white) and H3Cit (green). Bar 25µm. **(C)** Quantification of neutrophils in lungs from pristane-induced DAH WT and NLRP3 -/- mice. WT and NLRP3 -/- mice challenged with PBS IP injection were a negative control. Results represent median ± interquartile range, ANOVA multiple comparison test was used for statistical analysis between groups (ns: non-significant, *p*< 0.001^***^). Quantification of H3Cit in lungs from pristane-induced DAH WT and NLRP3 -/- mice. WT and NLRP3 -/- mice challenged with PBS IP injection were a negative control. Results represent median ± interquartile range, ANOVA multiple comparison test was applied for statistical analysis between groups (ns: non-significant, *p*< 0.01^**^, < 0.001^***^). Each experiment had n=3-6 mice/PBS group and n=7-10 mice/pristane group.

A single IP injection of pristane was shown to induce lung damage with neutrophil recruitment and NET formation in the lungs of WT mice (11). Using intravital microscopy, we confirmed neutrophil recruitment in the lungs of mice after pristane injection compared to lungs of PBS-injected mice (**Supplementary Figure 2**). Because NLRP3 deficiency reduced the severity of DAH in females (**Figure 2**), we determined whether NLRP3 deficiency could have an impact on neutrophil recruitment in BAL and the lungs of pristane-induced DAH mice (**Figures 4A–C**). Using fluorescence cytometry, we analyzed the neutrophil fraction in BAL. Among CD45+ cells, we detected a higher concentration of neutrophils (Ly6G+/Ly6C-) in BAL from pristane-induced DAH mice compared to BAL from PBS-injected mice (negative control) (**Figures 4B, C**). In female mice, the concentration of neutrophils was reduced in BAL from NLRP3-/-, compared with WT mice. In contrast, no difference was detected in BAL from NLRP3-/- male compared with WT males.

We then sought to determine the density of NETs (characterized by colocalization of DNA with extracellular citrullinated histones) in the lungs of pristane-induced DAH animals (**Figures 4B, C**). In female mice, the number of neutrophils (characterized by colocalization of Ly6G and DNA staining) in lungs was reduced in NLRP3-/- mice compared with WT while there was no difference in lungs of NLRP3-/- males compared with WT males. In addition, H3Cit fluorescence intensity was reduced in the lungs of NLRP3-/- females compared with WT females, while no difference in the lungs of males regardless of the NLRP3 status was detected. These results showed that NLRP3 deficiency reduced the number of neutrophils and NET formation in the lungs of pristane-induced DAH females, but not males.

## 4 Discussion

Our study provides evidence for a female sex-specific NLRP3 inflammasome activation contributing to the pathogenesis of pristane-induced DAH. We demonstrated *in vitro* that NLRP3 was needed for speck formation and NLRP3 inflammasome activation participated in pristane-induced NETosis independently of the sex. Interestingly, pristane-induced DAH was less severe in NLRP3-/- females, with less recruitment of neutrophils and NET generation in their lungs resulting in a significantly decreased pathological score, indicating the importance of female hormone environment for DAH development.

Pristane-induced DAH model is a limited organ disease that mimics pathological lesions of DAH related to SLE and SVV (11). The role of neutrophils in this model has been previously investigated. A study demonstrated the recruitment of neutrophils into the peritoneal cavity after IP pristane injection leading to chronic peritonitis (8). We previously reported the recruitment of neutrophils in lungs of pristane-induced DAH, with a less severe disease after deoxyribonuclease-1 therapy or neutrophil depletion, suggesting an important role of neutrophils and NETs in exacerbating this model (11). Pristane is considered to be an irritant with well-known adjuvant properties and subsequent cell cytotoxicity leading to apoptosis, necrosis, and, more recently observed, NETosis (11). Here, we showed that pristane induced the formation of NLRP3 inflammasome in neutrophils, subsequently supporting NETosis since we detected a significant reduction of ASC speck formation and NETosis in WT neutrophils pretreated with MCC950, a specific inhibitor of NLRP3 inflammasome and NLRP3-/- neutrophils, which is in line with a previous study published by our group, demonstrating significant reduction of NET formation in NLRP3-/- neutrophils after other kinds of sterile stimulation (6).

Genetic deletion of NLRP3 conferred a less severe DAH in females, but we did not detect any difference in males, suggesting a sex-specific NLRP3 inflammasome activation participating in the development of pristane-induced DAH. A sex-specific role of NLRP3 in diseases has been previously observed. NLRP3 deficiency reduced diet-induced atherosclerosis in female LDL-receptor deficient mice but not in males, a disease also highly dependent on leukocyte recruitment and IL-1β. Interestingly, this sex difference was lost after ovariectomy, suggesting a role for estrogen and/or progesterone in inflammasome-mediated atherogenesis (17). Although estrogens have well-known immune-enhancing properties, their role in regulating NLRP3 inflammasome activation is more complicated. Indeed, in contrast to our study, estrogens were reported to inhibit NLRP3 inflammasome pathway activation, caspase-1, and proinflammatory cytokine production in the brain after global cerebral ischemia, while activating NLRP3 inflammasome and triggering pyroptosis in hepatocellular carcinoma cells. Perhaps the effect of estrogens depends on the cell type in which inflammasome is activated (18, 19). In our study, *in vitro* levels of ASC speck formation and NETosis were similar between male and female neutrophils exposed to similar conditions, also suggesting the role of microenvironment *in vivo* in the activation of neutrophil NLRP3 inflammasome.

In addition, the severity of disease presentation may depend on other downstream events beyond NLRP3 inflammasome activation. A previous study showed that females were completely protected against the development of the disease in a murine model of transfusion-related acute lung injury that could be related to complement levels (20). However, in our study, the severity of DAH was similar between WT males and WT females.

We detected a reduced number of neutrophils and NETs in lungs of pristane-induced DAH NLRP3-/- females compared to males, suggesting a link between NLRP3 inflammasome activation and neutrophil recruitment. In 2012, NLRP3 inflammasome was reported to promote immune cell migration to the central nervous system in an experimental murine autoimmune encephalomyelitis model, mediated by myelin-specific autoreactive T-Helper cells (21). In addition, we previously demonstrated that the formation of NLRP3 inflammasome at the neutrophil microtubule organizing center was necessary for neutrophil polarization and directional migration toward an *in vitro* gradient of the chemoattractant leukotriene B4 and *in vivo* to a laser-induced liver burn injury (22). More recently, our group showed that neutrophil NLRP3 promoted the recruitment of neutrophils to the myocardium and the release of NETs in myocardium during a myocardial infarction murine model (23). Combining our observations with these data, we also suspect a defect in the migration system of neutrophils during pristane-induced DAH in NLRP3-/- females. The reduced number of NLRP3-induced NETs likely limits the development of DAH lesions, since we previously demonstrated a negative impact of NETs on pristane-induced DAH.

It is important to mention that another study found no substantial impact of neutrophil depletion in the development of pristane-induced DAH, but rather that monocytes were key (24). In this study, sex of mice was not considered. Indeed, monocyte NLRP3 maybe also implicated in the cytokine release and subsequent leukocyte recruitment as previously described in a murine model of gout (25). In addition, a study demonstrated the opsonization of lung dead cells by natural IgM and complement followed by complement receptor-mediated lung inflammation that could contribute to the development of pristane-induced DAH (10).

Our study has limitations. We used the pristane-induced DAH model that is not considered as an exclusive autoimmune model of DAH, where a direct toxic effect of pristane causing lung injury could participate. However, the clinical presentation in mice associated with pathological lesions are similar to DAH-related SLE and SVV. Ours is an *in vivo* pilot study with promising results about the sex-specific activation of NLRP3 inflammasome in neutrophils promoting pulmonary NETosis during the pristane-induced DAH model. We cannot exclude that the reduction of neutrophil infiltration may affect NETosis in NLRP3-/- females, further experiments are needed to confirm whether NLRP3 inflammasome directly regulate the effect of NETosis in this model, and test NLRP3 inflammasome inhibitors, such as MCC950 in this model. It will be also important to examine whether females hormones regulate PAD4, NLRP3, or ASC protein levels, all important for efficient inflammasome assembly (6).

In conclusion, our data indicate a more significant effect of NLRP3 activation in the females during pristane-induced DAH. The reduced number of neutrophils and NETs in lungs of NLRP3-/- females suggest a link between NLRP3 inflammasome activation, neutrophil recruitment, and NET participation in the disease process. These observations are of a great importance since females are often of an increased risk for autoimmune disease and we added promising results in identifying neutrophil NLRP3 inflammasome as a potential interesting target, with specific NLRP3 inflammasome inhibitors currently in development.

## Data Availability

The raw data supporting the conclusions of this article will be made available by the authors, without undue reservation.

## Supplementary material

Supplementary Figure 1 Severity of DAH is similar between WT males and WT females **(A)** Oxygen-saturated hemoglobin level (%) in mice with pristane-induced DAH WT males and WT females. Results represent median ± interquartile range, Mann Whitney test was used for statistical analysis to compare the two groups (ns: non-significant). **(B)** DAH score from pristane-induced DAH WT and NLRP3 -/- mice. Results represent median ± interquartile range, Mann Whitney test was used for statistical analysis to compare the two groups (ns: non-significant). Each experiment had n=7-10 mice/pristane group.

Supplementary Figure 2 NLRP3 deficiency and severity of pristane-induced DAH **(A, B)** Left column shows results with male mice, right with female mice. **(A)** Quantification of maximum weight loss from pristane-induced DAH WT and NLRP3 -/- mice. Results represent median ± interquartile range, Mann Whitney test was used for statistical analysis to compare the two groups (ns: non-significant). **(B)** Quantification of wet/dry (W/D) ratio from pristane-induced DAH WT and NLRP3 -/- mice. WT and NLRP3-/- mice challenged with PBS IP injection were a negative control. Results represent median ± interquartile range, ANOVA multiple comparison test was applied for statistical analysis between groups (ns: non-significant). Each experiment had n=3-6 mice/PBS group and n=7-10 mice/pristane group.

Supplementary Figure 3 Recruitment of neutrophils in lungs of pristane-induced DAH **(A)** Representative intravital microscopy images acquired every second for a period of 7 min. Blood vessels are in red (Dextran rhodamine B), neutrophils are in blue (Ly6G BV 421, white arrowhead). Bar: 30μm. **(B)** Quantification of neutrophils in lungs from pristane-induced DAH WT and WT mice challenged with PBS IP injection (negative control). Results represent median ± interquartile range, Mann Whitney test was used for statistical analysis to compare the two groups (*p* < 0.001^***^). Three male mice were evaluated in pristane-treated group and in PBS-injected mice (negative control).
