# Supplementary figures and images for: Sex-specific NLRP3 activation in neutrophils promotes neutrophil recruitment and NETosis in the murine model of diffuse alveolar hemorrhage
Source: Front Immunol. 2024 Nov 25;15:1466234. doi: 10.3389/fimmu.2024.1466234 (PMC11625668; doi:10.3389/fimmu.2024.1466234)

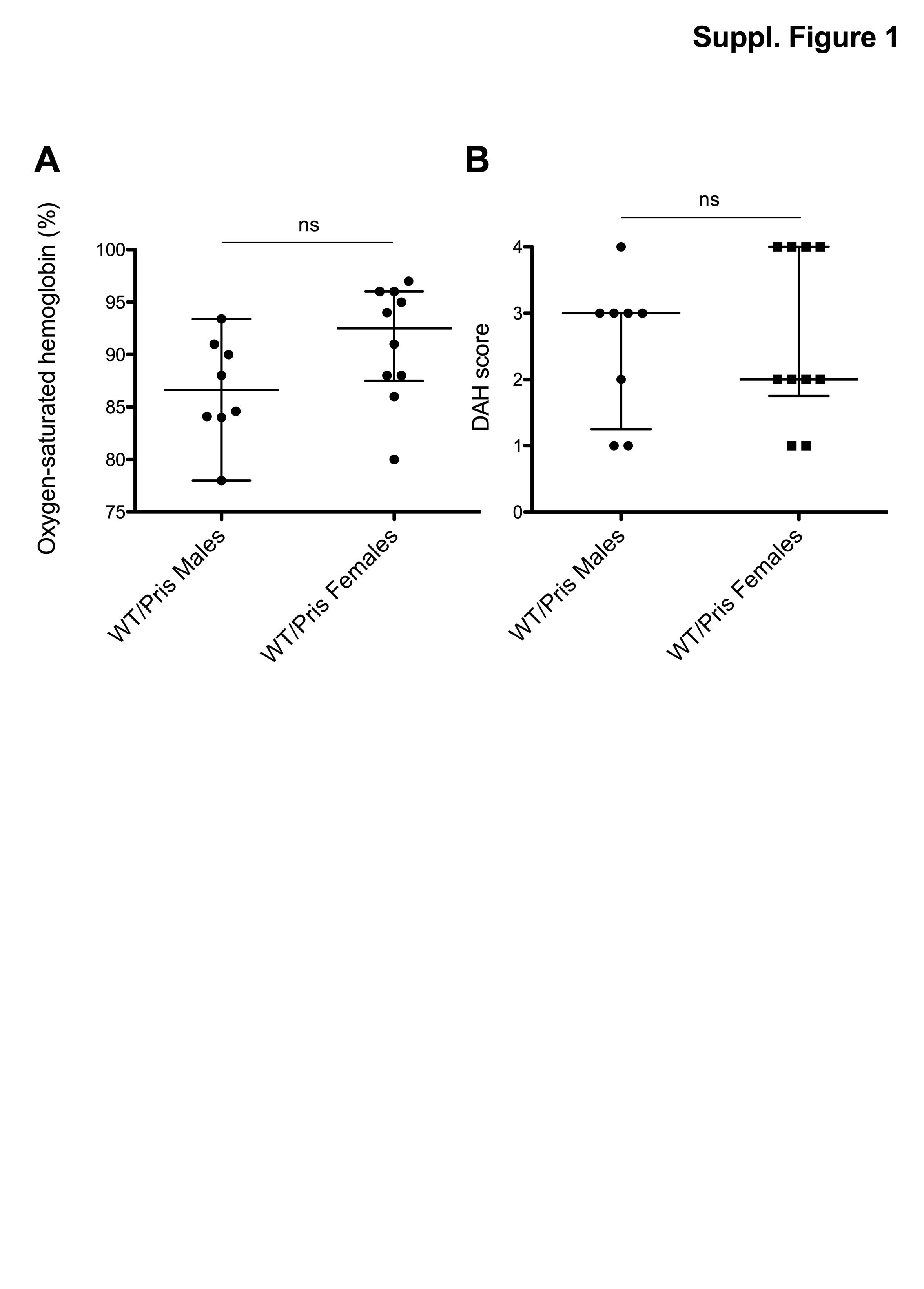

Supplement: Supplementary Figure 1 — Severity of DAH is similar between WT males and WT females (A) Oxygen-saturated hemoglobin level (%) in mice with pristane-induced DAH WT males and WT females. Results represent median ± interquartile range, Mann Whitney test was used for statistical analysis to compare the two groups (ns: non-significant). (B) DAH score from pristane-induced DAH WT and NLRP3 -/- mice. Results represent median ± interquartile range, Mann Whitney test was used for statistical analysis to compare the two groups (ns: non-significant). Each experiment had n=7-10 mice/pristane group. [file Image1.jpeg]

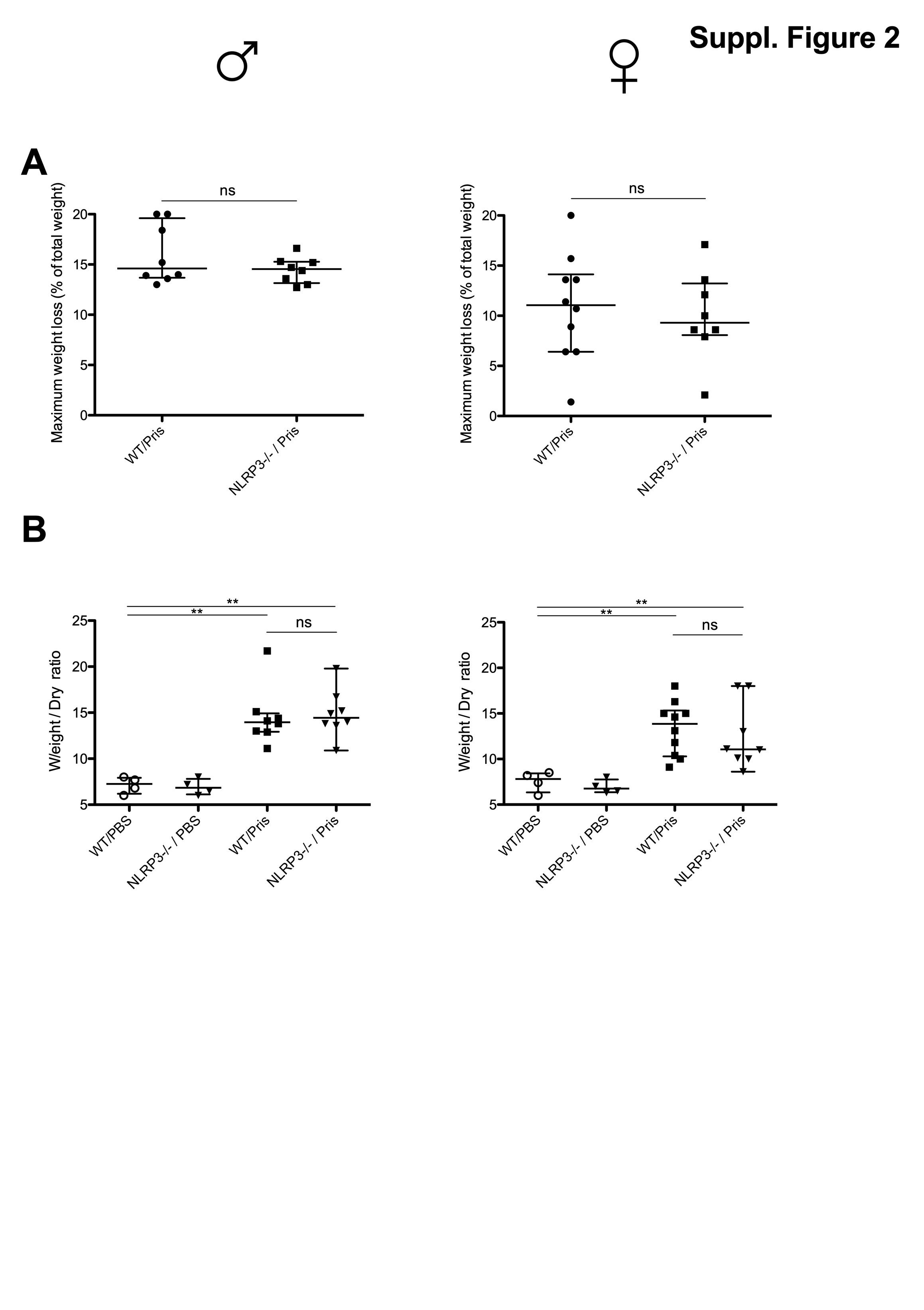

Supplement: Supplementary Figure 2 — NLRP3 deficiency and severity of pristane-induced DAH (A, B) Left column shows results with male mice, right with female mice. (A) Quantification of maximum weight loss from pristane-induced DAH WT and NLRP3 -/- mice. Results represent median ± interquartile range, Mann Whitney test was used for statistical analysis to compare the two groups (ns: non-significant). (B) Quantification of wet/dry (W/D) ratio from pristane-induced DAH WT and NLRP3 -/- mice. WT and NLRP3-/- mice challenged with PBS IP injection were a negative control. Results represent median ± interquartile range, ANOVA multiple comparison test was applied for statistical analysis between groups (ns: non-significant). Each experiment had n=3-6 mice/PBS group and n=7-10 mice/pristane group. [file Image2.jpeg]

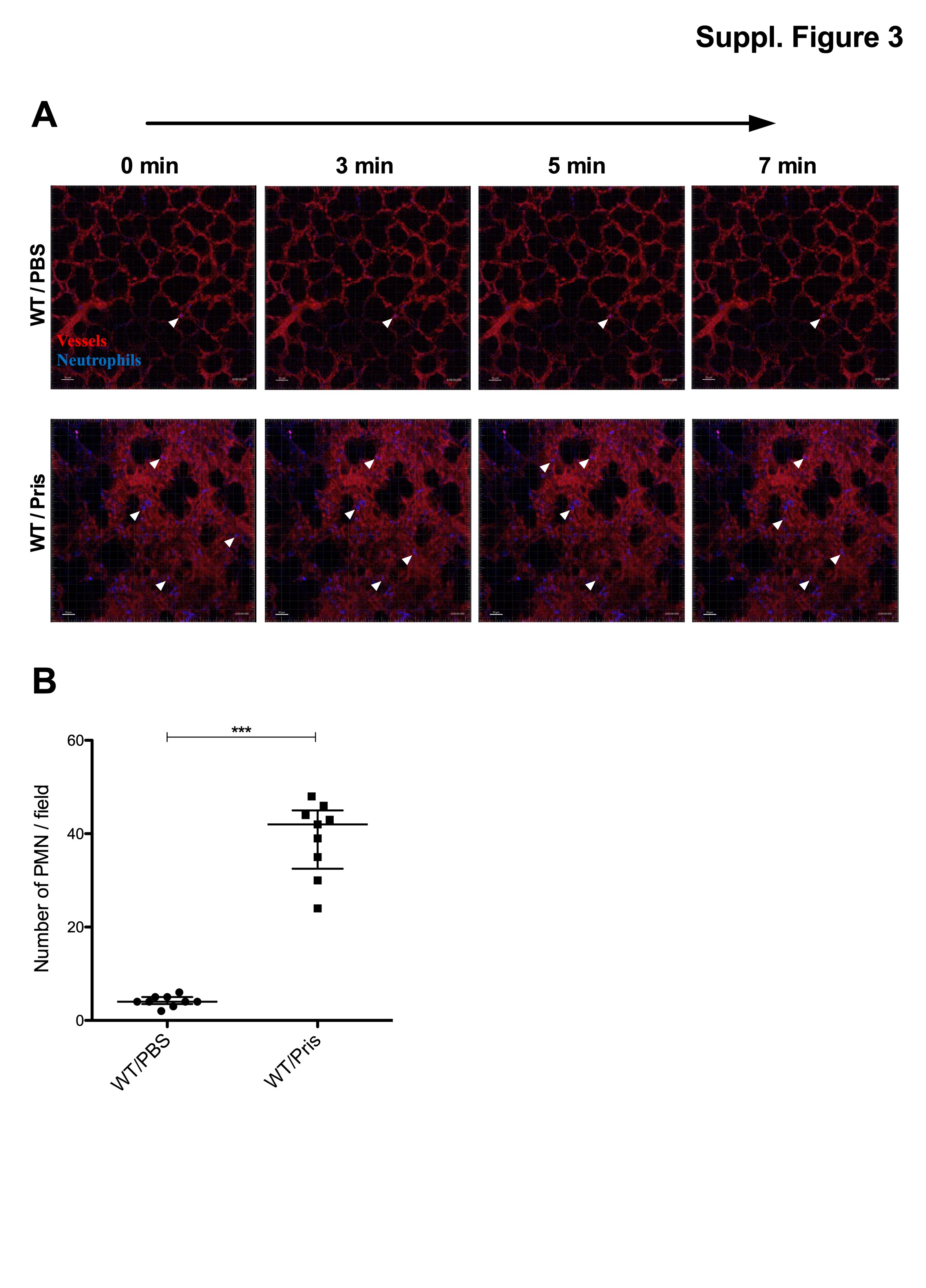

Supplement: Supplementary Figure 3 — Recruitment of neutrophils in lungs of pristane-induced DAH (A) Representative intravital microscopy images acquired every second for a period of 7 min. Blood vessels are in red (Dextran rhodamine B), neutrophils are in blue (Ly6G BV 421, white arrowhead). Bar: 30μm. (B) Quantification of neutrophils in lungs from pristane-induced DAH WT and WT mice challenged with PBS IP injection (negative control). Results represent median ± interquartile range, Mann Whitney test was used for statistical analysis to compare the two groups (p < 0.001***). Three male mice were evaluated in pristane-treated group and in PBS-injected mice (negative control). [file Image3.jpeg]
